# Supplementary figures and images for: A generic cell surface ligand system for studying cell–cell recognition
Source: PLoS Biol. 2019 Dec 9;17(12):e3000549. doi: 10.1371/journal.pbio.3000549 (PMC6922461; doi:10.1371/journal.pbio.3000549)

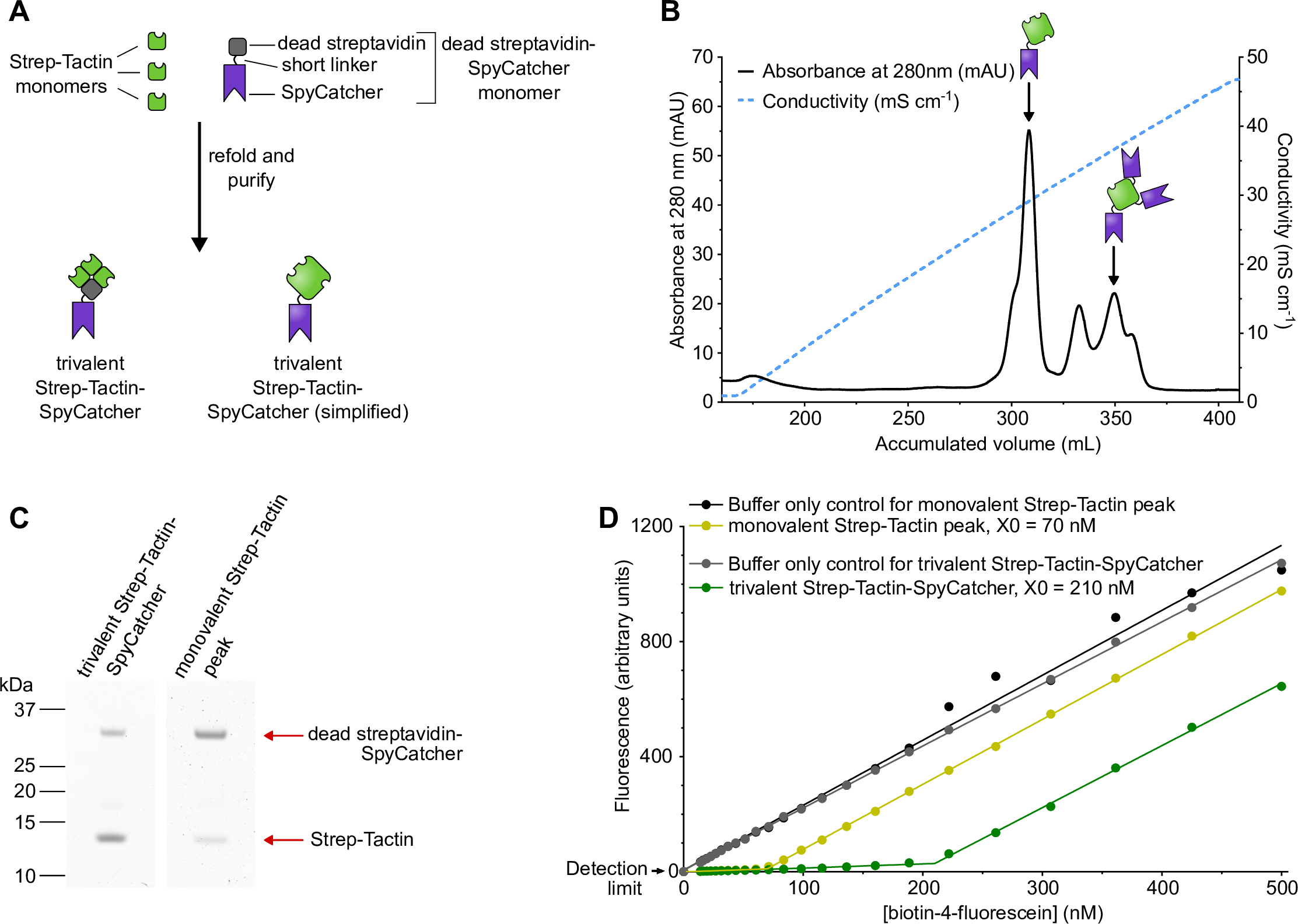

Supplement: S1 Fig — (A) Trivalent Strep-Tactin-SpyCatcher is synthesised by refolding mixtures of bacterially produced Strep-Tactin and dead streptavidin-SpyCatcher monomers in a 3:1 ratio. This desired tetramer is shown in schematic form alongside a more simplified cartoon. (B) An anion exchange chromatogram showing elution of the predicted trivalent Strep-Tactin-SpyCatcher peak alongside other configurations including a monovalent Strep-Tactin tetramer with a single Strep-tag II–binding site and three SpyCatchers. (C) SDS-PAGE analysis of the eluted anion exchange chromatography peaks predicted to contain trivalent Strep-Tactin-SpyCatcher and monovalent Strep-Tactin. The samples were boiled prior to loading, and the gel was stained with Coomassie to show the relative proportion of subunits. Densitometry was performed on Strep-Tactin and dead streptavidin-SpyCatcher bands, and the values were normalised for subunit molecular weights and then converted into a Strep-Tactin:dead streptavidin-SpyCatcher subunit ratio. Trivalent Strep-Tactin-SpyCatcher = 4.7:1 (expected 3:1), monovalent Strep-Tactin peak = 0.6:1 (expected 0.33:1). (D) Trivalent Strep-Tactin-SpyCatcher or the predicted monovalent peak for comparison (50 nM) was incubated with a titration of biotin-4-fluorescein in a fluorescence-quenching assay. Inflection point X values (X0) are shown. Summary numerical data are provided in S1 Data; original gel images are provided in S1 Raw images. (TIF) [file pbio.3000549.s001.tif]

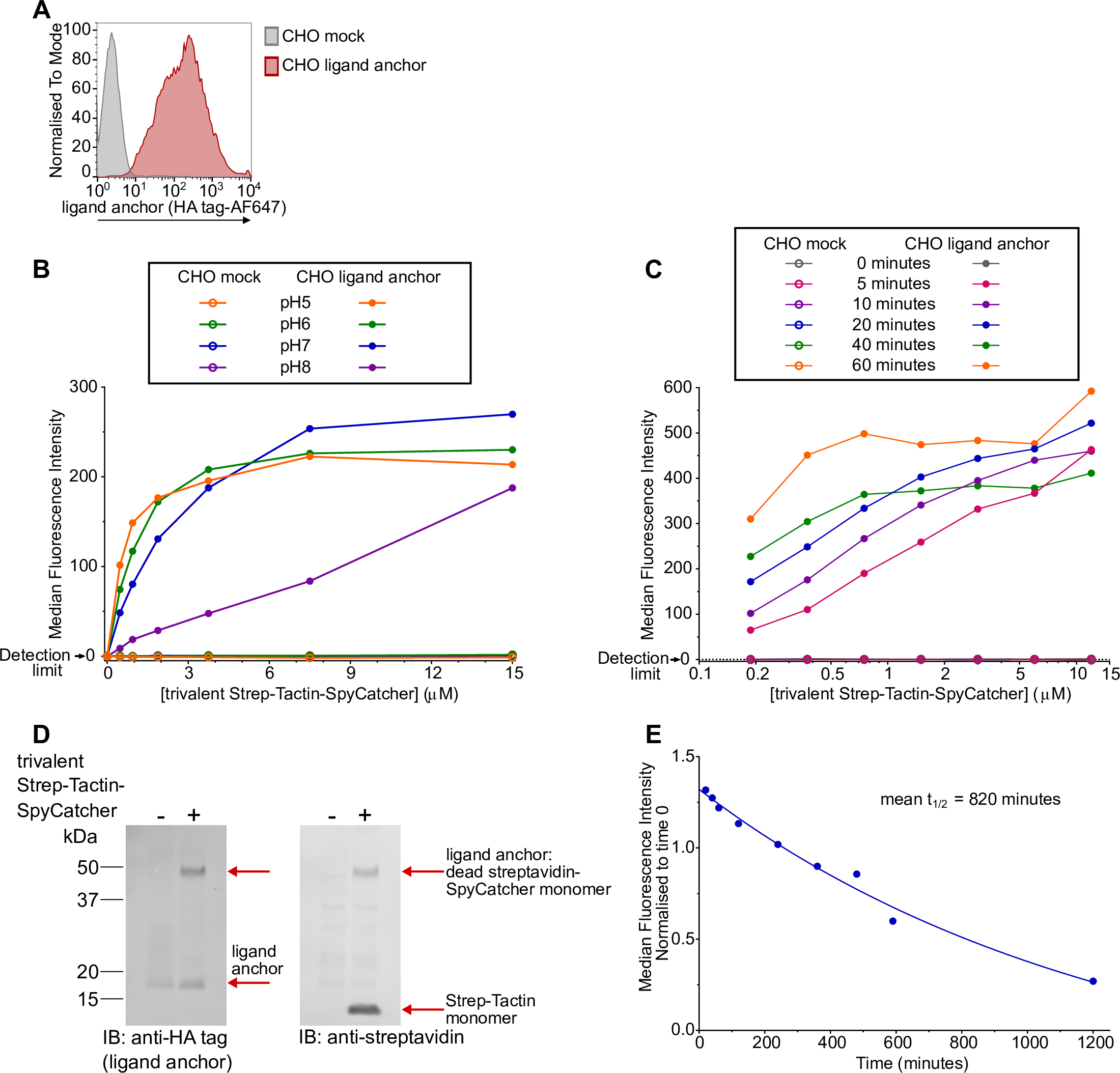

Supplement: S2 Fig — (A) Ligand anchor expression by transfected CHO cells as determined using antibody to N-terminal HA tag and flow cytometry (CHO mock: mock transfected). The efficiency of CHO ligand anchor:trivalent Strep-Tactin-SpyCatcher coupling at 25°C under different pH conditions (B) or with varying cell–protein incubation times before washing (C) is shown. Cells were incubated with ATTO 647 biotin to indicate generic ligand levels. MFI values, extracted from flow cytometry analyses, are shown as a function of trivalent Strep-Tactin-SpyCatcher concentration. (D) Ligand anchor:trivalent Strep-Tactin-SpyCatcher binding is covalent. Boiled lysates of CHO ligand anchor cells preincubated with trivalent Strep-Tactin-SpyCatcher or buffer only were analysed by western blotting. The Strep-Tactin-SpyCatcher tetramer dissociates upon boiling, and so the ligand anchor is visualised coupled to dead streptavidin-SpyCatcher subunit only. (E) Cell surface down-regulation of the generic ligand over time following reconstitution is visualised using ATTO 647 biotin. MFIs, extracted from flow cytometry analyses, are shown normalised to the MFI at time 0, which was given a value of 1. The mean half-life from two independent experiments (range = 780–860 minutes, n = 2) is shown. The generic ligand cell surface levels appear to rise within the first 20 minutes post-reconstitution, visualised as an increase in MFI. This may reflect a proportion of trivalent Strep-Tactin-SpyCatcher that is in contact with, but not yet covalently bound to, ligand anchor during the initial incubation and so is removed during the process of analysing ligand cell surface levels. Incubating the cells at 37°C post-reconstitution may allow this proportion of protein to covalently, irreversibly bind to the ligand anchor and thereby lead to an apparent increase in cell surface levels. Summary numerical data are provided in S1 Data; gating strategy and original .fcs files are provided in S2 Data; original gel images are provid [file pbio.3000549.s002.tif]

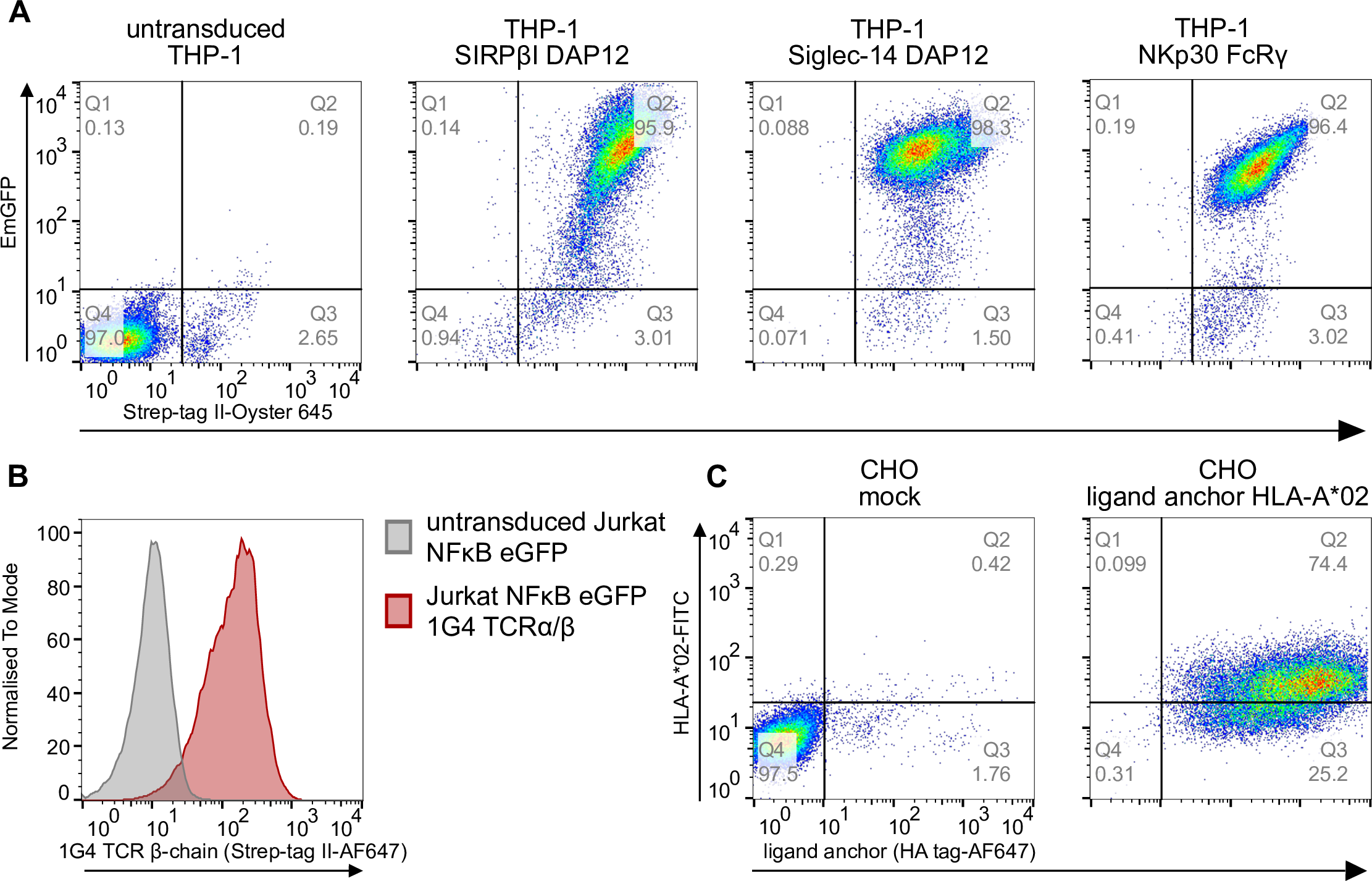

Supplement: S3 Fig — (A) Expression of one of three Twin-Strep-tagged receptors and appropriate adaptor by THP-1 cells. Using flow cytometry, receptor expression was analysed using anti-Strep-tag II antibody. Expression of the exogenous, introduced adaptor was inferred using an IRES-EmGFP sequence. (B) Expression of 1G4 TCRα/β-Twin-Strep-tag by Jurkat NFκB eGFP cells as shown using anti-Strep-tag II antibody and flow cytometry. (C) Expression of the generic ligand anchor and HLA-A*02 SCD by CHO cells shown using anti-HA tag antibody and anti-HLA-A*02 antibody, respectively. Numbers indicate percentage of events in each quadrant. Gating strategy and original .fcs files in S2 Data. CHO, Chinese hamster ovary; eGFP, enhanced green fluorescent protein; HA, hemagglutinin; IRES-EmGFP, internal ribosome entry site–emerald green fluorescent protein; NFκB, nuclear factor kappa-light-chain-enhancer of activated B cells; SCD, single-chain dimer; TCR, T-cell receptor. (TIF) [file pbio.3000549.s003.tif]

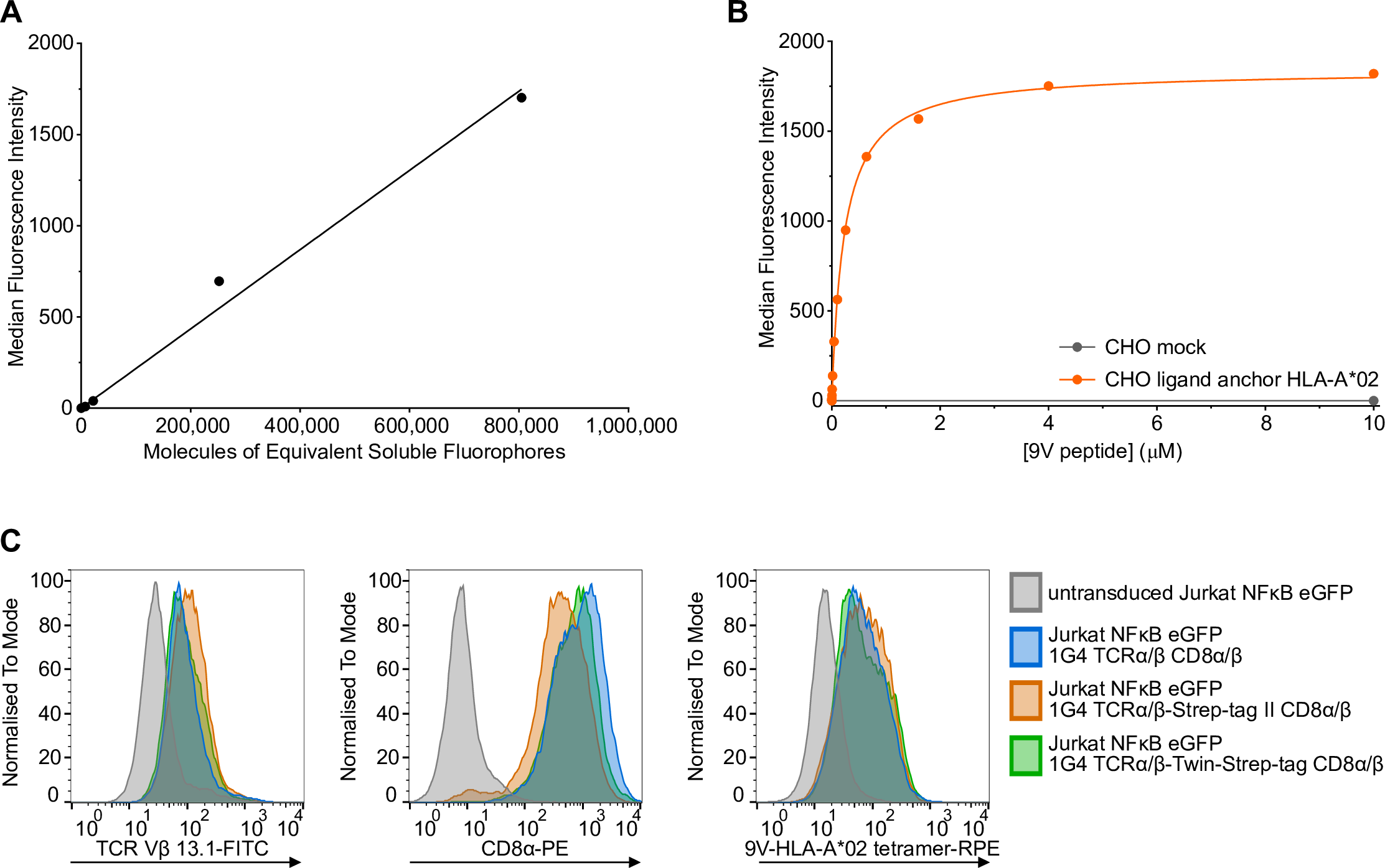

Supplement: S4 Fig — (A) Median fluorescence intensity values from flow cytometry analysis of Alexa Fluor 647 fluorescence quantitation beads used to create a standard curve. (B) A relative indication of the level of 9V-HLA-A*02 per cell as a function of 9V peptide concentration added to cells. Median fluorescence intensity values extracted from flow cytometry analyses of cells incubated with soluble 1G4 high-affinity TCRα/β Alexa Fluor 647 are shown. (C) Jurkat reporter cells expressing CD8α and β and 1G4 TCRα/β either nontagged or tagged with Strep-tag II or Twin-Strep-tag show comparable levels of TCRβ chain (left) and CD8α (centre) expression and 9V-HLA-A*02 tetramer binding (right). Summary numerical data are provided in S1 Data; gating strategy and original .fcs files are in S2 Data. TCR, T-cell receptor. (TIF) [file pbio.3000549.s004.tif]

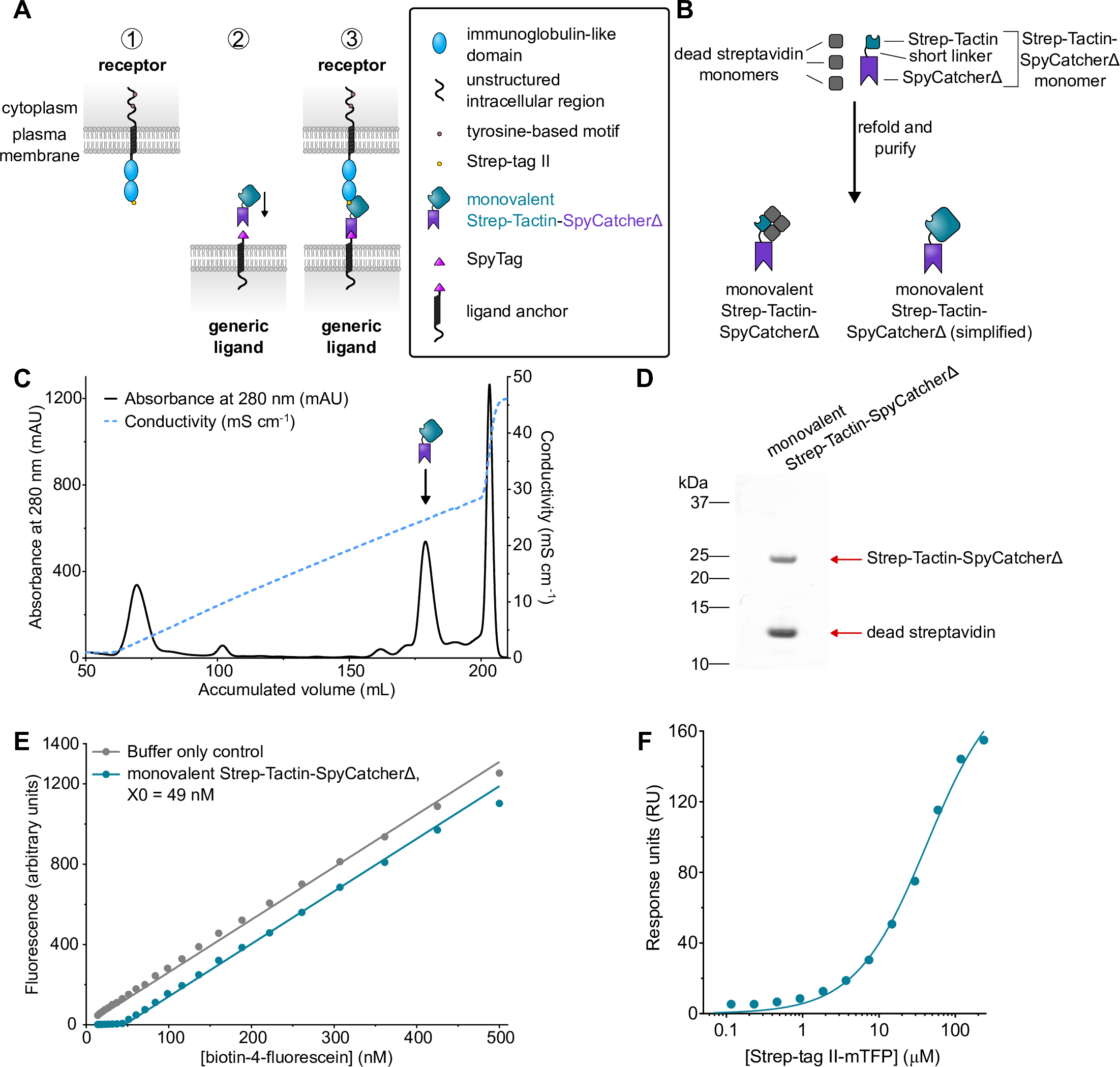

Supplement: S5 Fig — (A) (1) The receptor is constructed with N-terminal Strep-tag II. (2) Soluble monovalent Strep-Tactin-SpyCatcherΔ protein covalently binds to the generic ligand anchor. (3) The single binding site of monovalent Strep-Tactin-SpyCatcherΔ is available for ligation by the Strep-tag II–tagged receptor. (B) Monovalent Strep-Tactin-SpyCatcherΔ is synthesised by refolding mixtures of bacterially produced Strep-Tactin-SpyCatcherΔ and dead streptavidin monomers in a 1:3 ratio. (C) An anion exchange chromatogram showing elution of the predicted monovalent Strep-Tactin-SpyCatcherΔ peak alongside other configurations. (D) SDS-PAGE analysis of the eluted anion exchange chromatography peak predicted to contain monovalent Strep-Tactin-SpyCatcherΔ. Densitometry was performed on the bands, and the values were normalised for subunit molecular weights and then converted into a Strep-Tactin-SpyCatcherΔ:dead streptavidin subunit ratio (1:3.7, expected 1:3). (E) Monovalent Strep-Tactin-SpyCatcherΔ (50 nM) was incubated with a titration of biotin-4-fluorescein in a fluorescence-quenching assay. The inflection point (X0) is shown. (F) Representative equilibrium binding from surface plasmon resonance of Strep-tag II–mTFP flown over immobilised monovalent Strep-Tactin-SpyCatcherΔ at 37°C. The KD (SEM) for the collated data from three independent experiments with two flow cells per experiment is 43 μM (4.5 μM), and the mean Hill slope (SEM) is 0.97 (0.085) to 2 s.f. Summary numerical data are provided in S1 Data; original gel images are provided in S1 Raw images. mTFP, monomeric teal fluorescent protein; SEM, standard error of the mean; s.f., significant figures. (TIF) [file pbio.3000549.s005.tif]

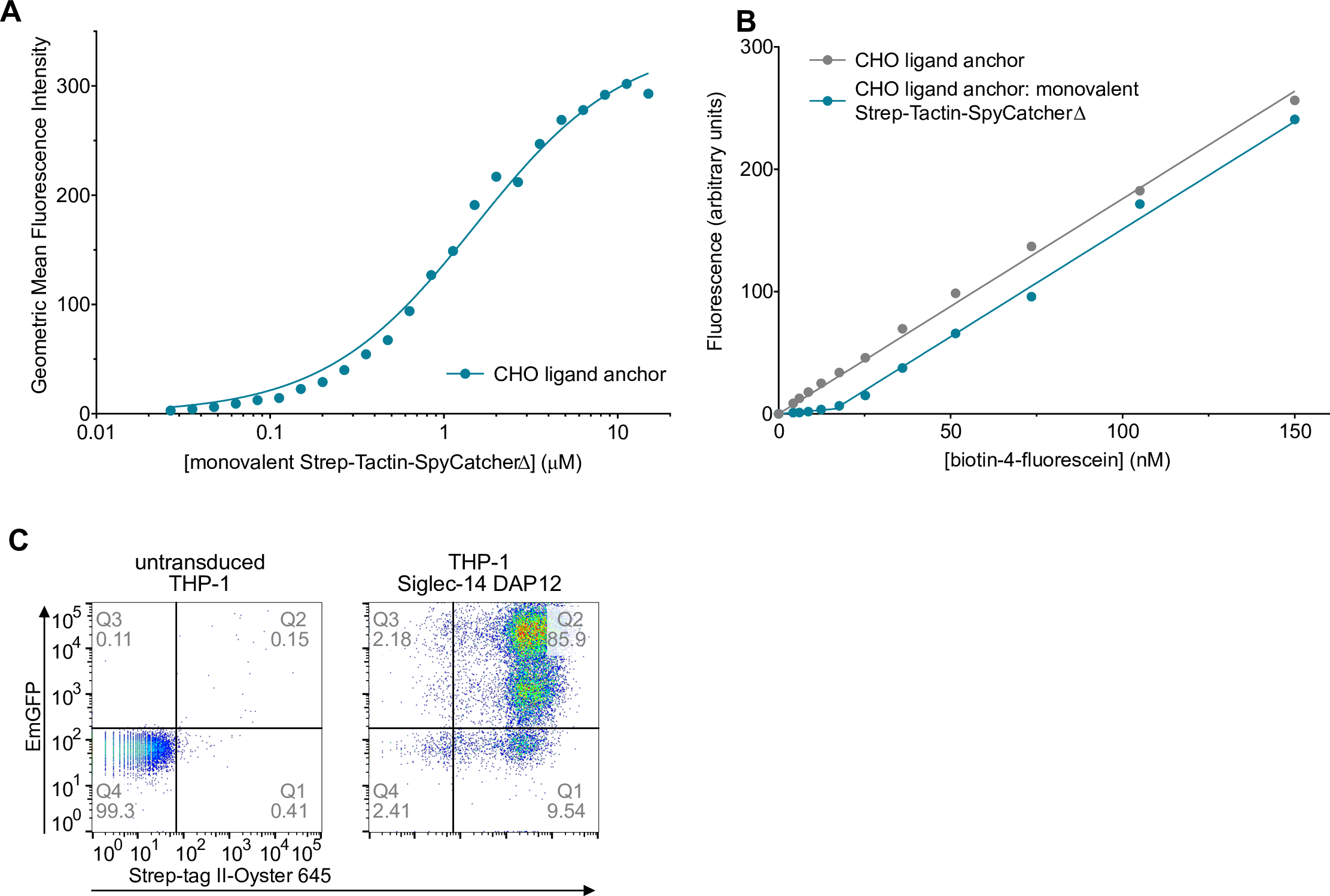

Supplement: S6 Fig — (A) A relative indication of the level of generic ligand per cell as a function of monovalent Strep-Tactin-SpyCatcherΔ concentration. Geometric mean fluorescence intensity values from flow cytometry analyses of cells incubated with ATTO 488 biotin are shown. (B) CHO ligand anchor cells preincubated with monovalent Strep-Tactin-SpyCatcherΔ or buffer alone were incubated with a titration of biotin-4-fluorescein in a fluorescence-quenching assay. (C) Expression of Siglec-14-Strep-tag II and exogenous DAP12 by THP-1 cells using anti-Strep-tag II antibody and an IRES-EmGFP sequence respectively and flow cytometry. Percentages of events in each quadrant are shown. Summary numerical data are provided in S1 Data; gating strategy and original .fcs files in S2 Data. CHO, Chinese hamster ovary; DAP12, DNAX-activating protein of 12 kDa; IRES-EmGFP, internal ribosome entry site–emerald green fluorescent protein; Siglec-14, Sialic acid–binding immunoglobulin-type lectin 14. (TIF) [file pbio.3000549.s006.tif]

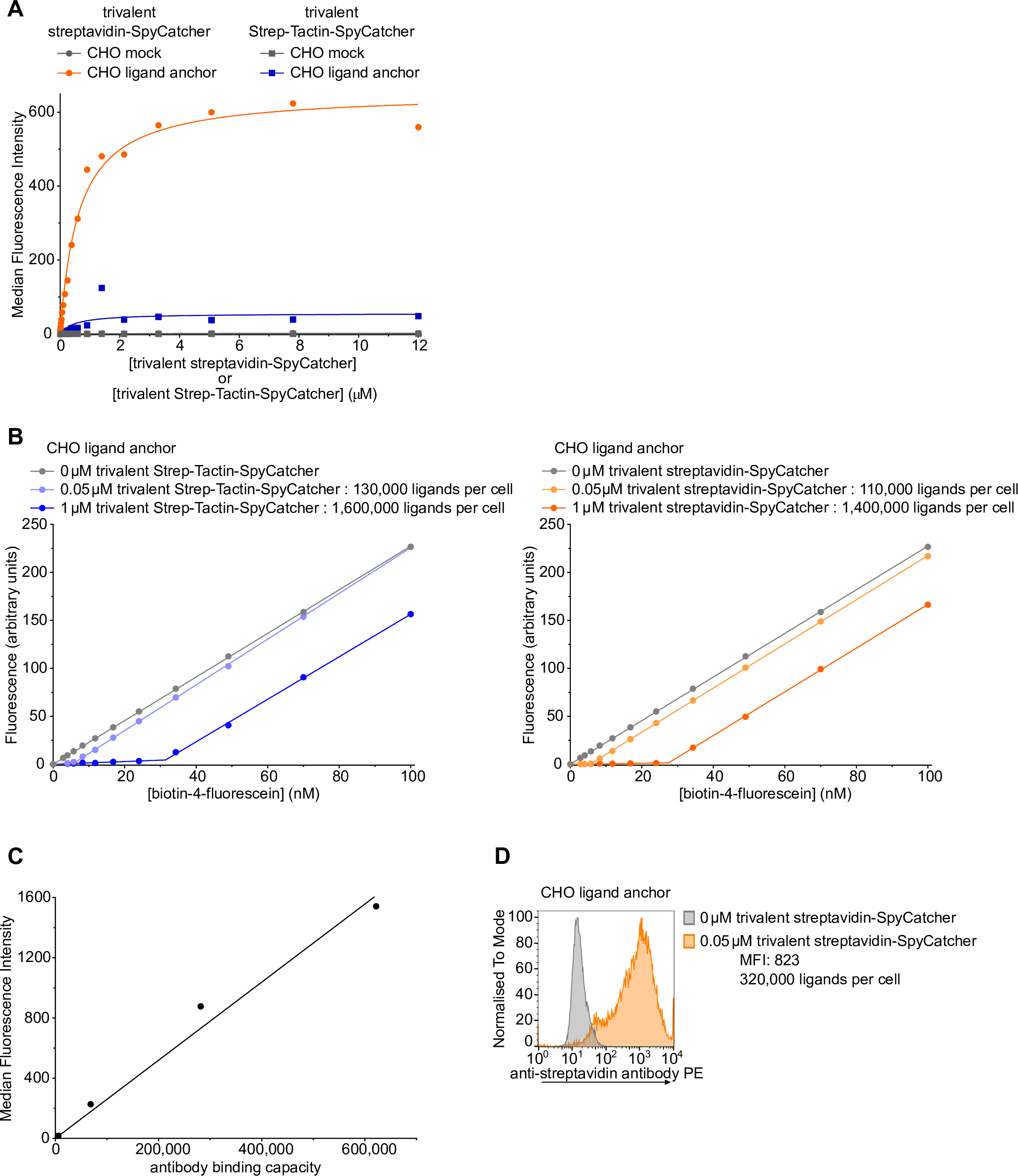

Supplement: S7 Fig — (A) CHO ligand anchor cells presenting either trivalent streptavidin-SpyCatcher or trivalent Strep-Tactin-SpyCatcher were analysed for anti-streptavidin antibody binding. Median fluorescence intensities from flow cytometry analyses are shown. (B) CHO ligand anchor cells preincubated with trivalent Strep-Tactin-SpyCatcher (left) or trivalent streptavidin-SpyCatcher (right) were incubated with a titration of biotin-4-fluorescein in a fluorescence-quenching assay. The average numbers of generic ligands per cell calculated using the X0 values are shown. (C) Anti-mouse IgG quantitation beads were incubated with anti-streptavidin antibody. Median fluorescence intensities from flow cytometry analyses are shown plotted against the antibody binding capacity of the beads. (D) CHO ligand anchor cells preincubated with trivalent streptavidin-SpyCatcher or buffer alone were incubated with anti-streptavidin antibody in parallel with the anti-mouse IgG beads in (C) and analysed by flow cytometry. The median fluorescence intensity value is shown alongside the number of ligands per cell calculated using this value and the standard curve in (C). Ligand numbers are given to 2 s.f. Summary numerical data are provided in S1 Data; gating strategy and original .fcs files are in S2 Data. CHO, Chinese hamster ovary; IgG, immunoglobulin G; s.f., significant figures. (TIF) [file pbio.3000549.s007.tif]

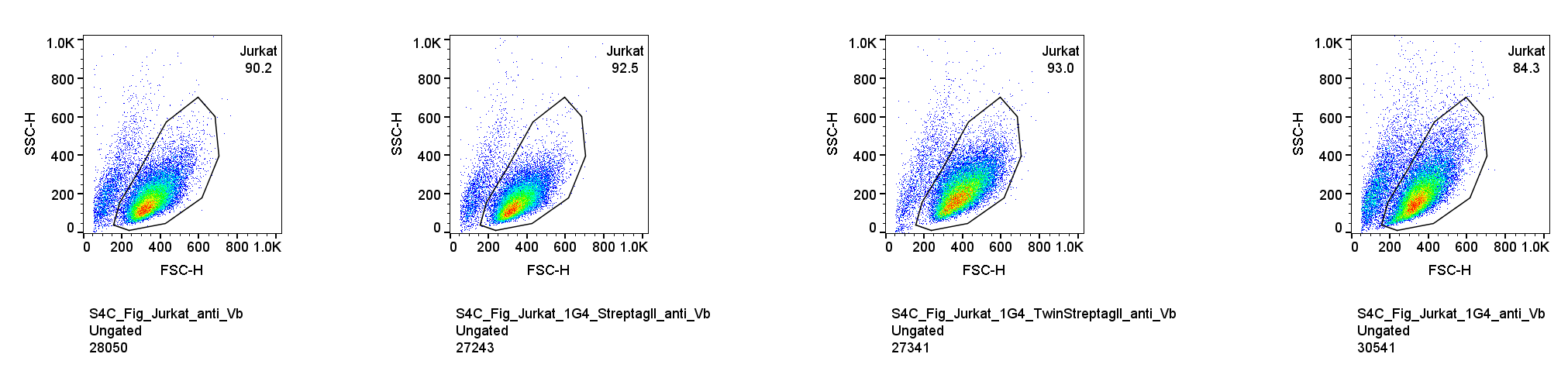

Supplement: S2 Data — (ZIP) [file pbio.3000549.s009.zip › S2_Data/S4C_Fig_anti_Vb_Gating_strategy.tiff]

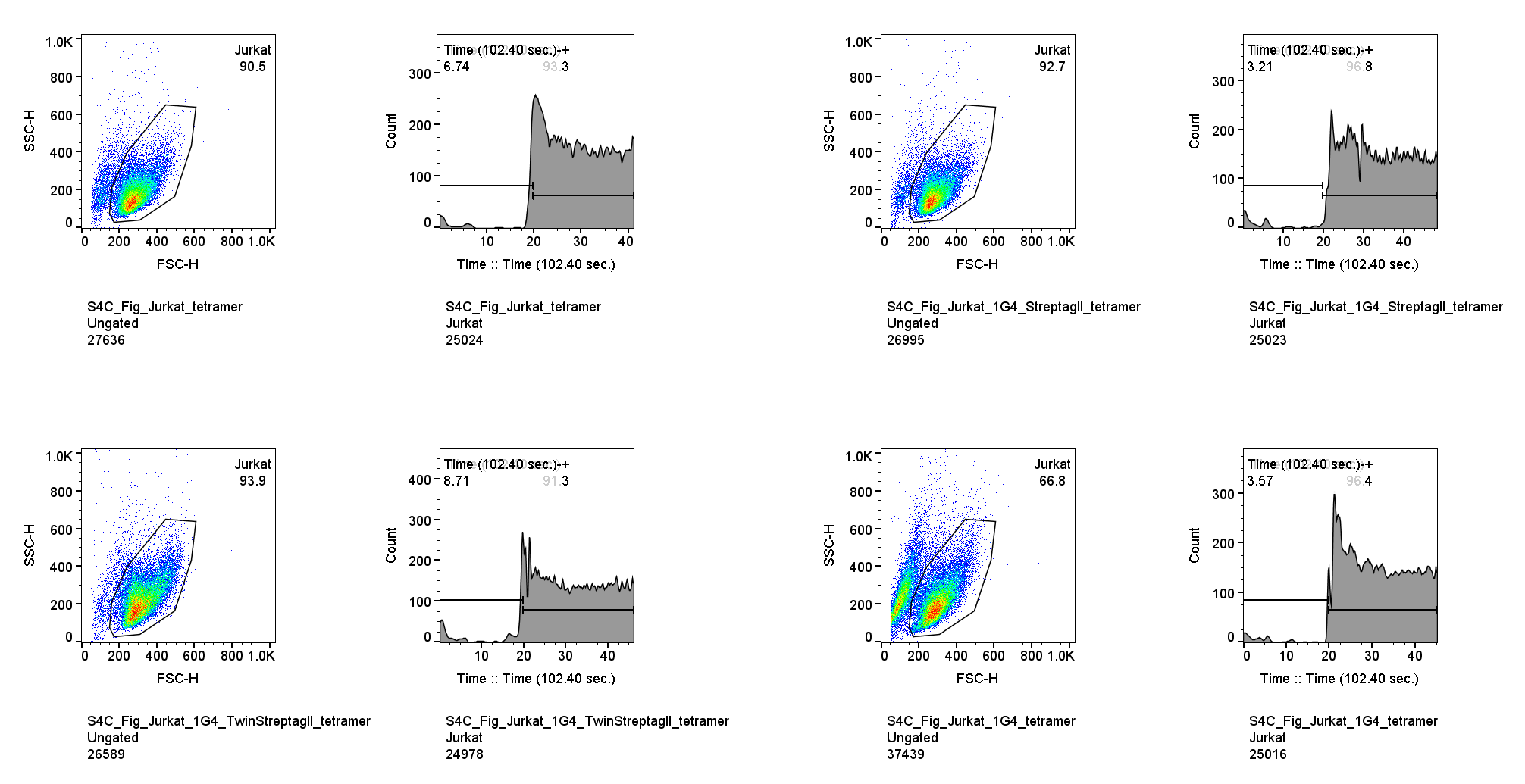

Supplement: S2 Data — (ZIP) [file pbio.3000549.s009.zip › S2_Data/S4C_Fig_tetramer_Gating_strategy.tiff]

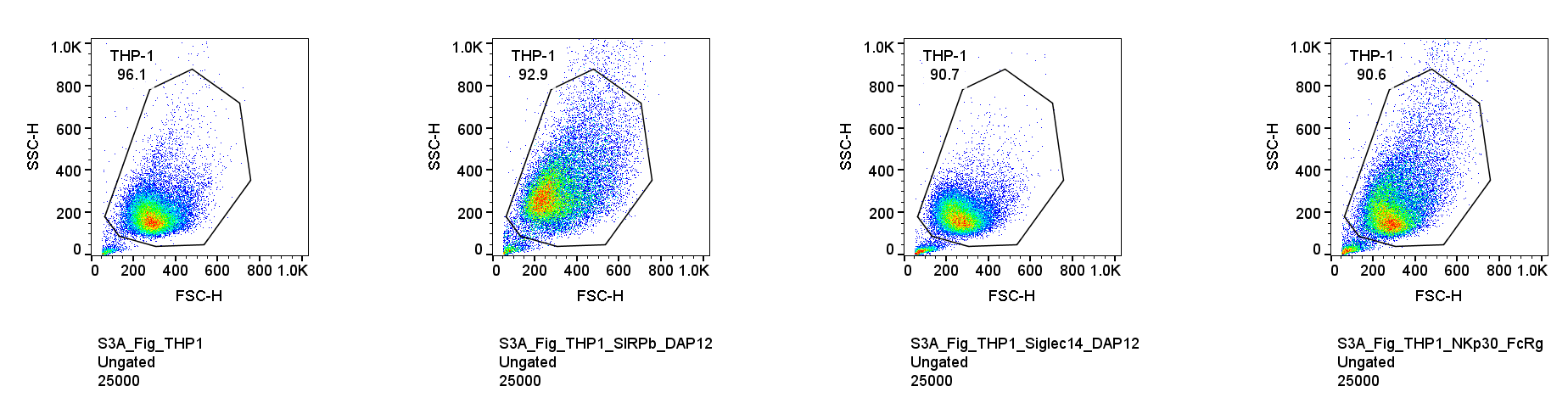

Supplement: S2 Data — (ZIP) [file pbio.3000549.s009.zip › S2_Data/S3A_Fig_Gating_strategy.tiff]

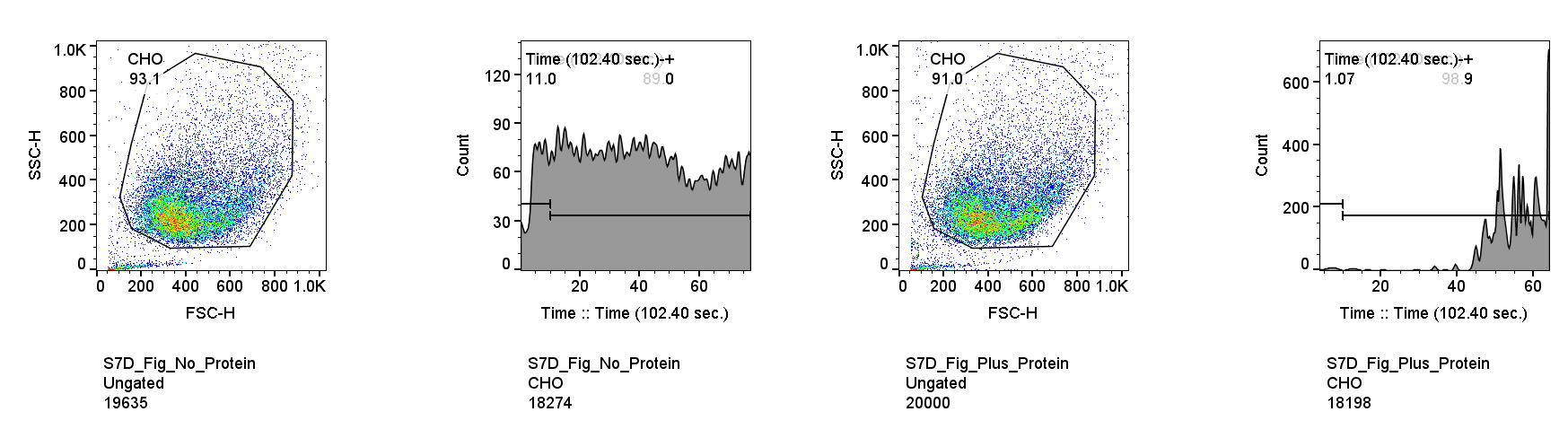

Supplement: S2 Data — (ZIP) [file pbio.3000549.s009.zip › S2_Data/S7D_Fig_Gating_strategy.tiff]

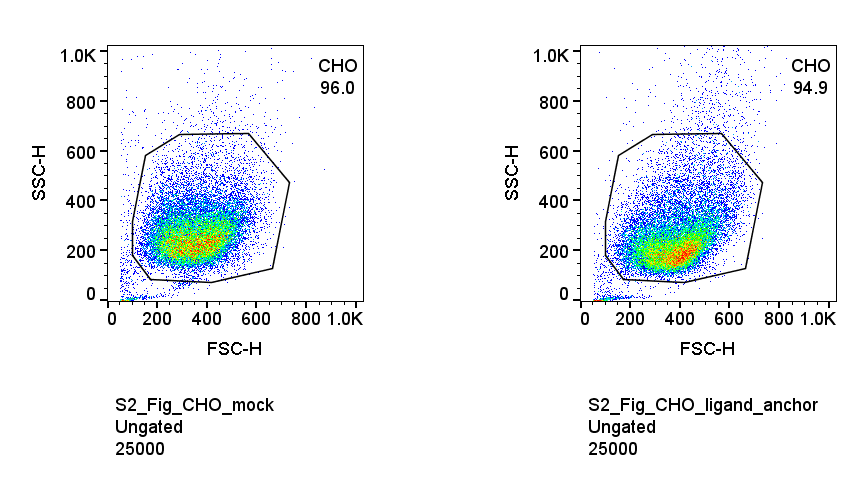

Supplement: S2 Data — (ZIP) [file pbio.3000549.s009.zip › S2_Data/S2A_Fig_Gating_strategy.tiff]

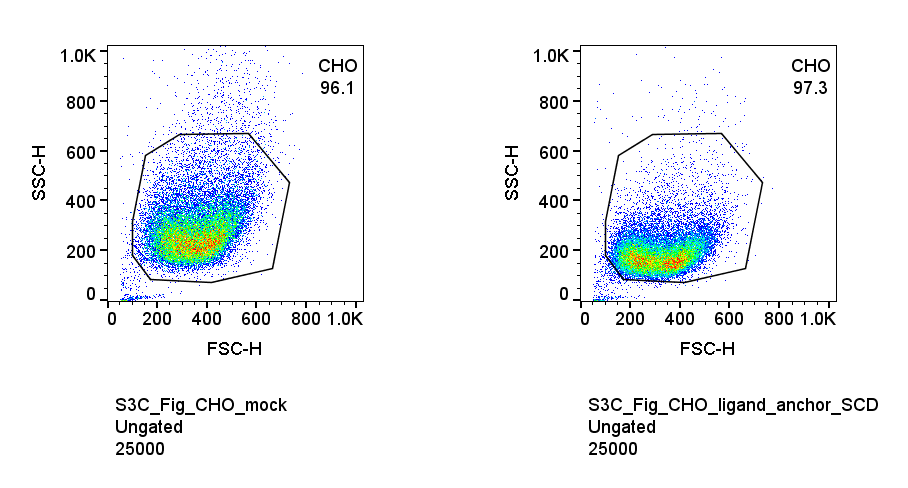

Supplement: S2 Data — (ZIP) [file pbio.3000549.s009.zip › S2_Data/S3C_Fig_Gating_strategy.tiff]

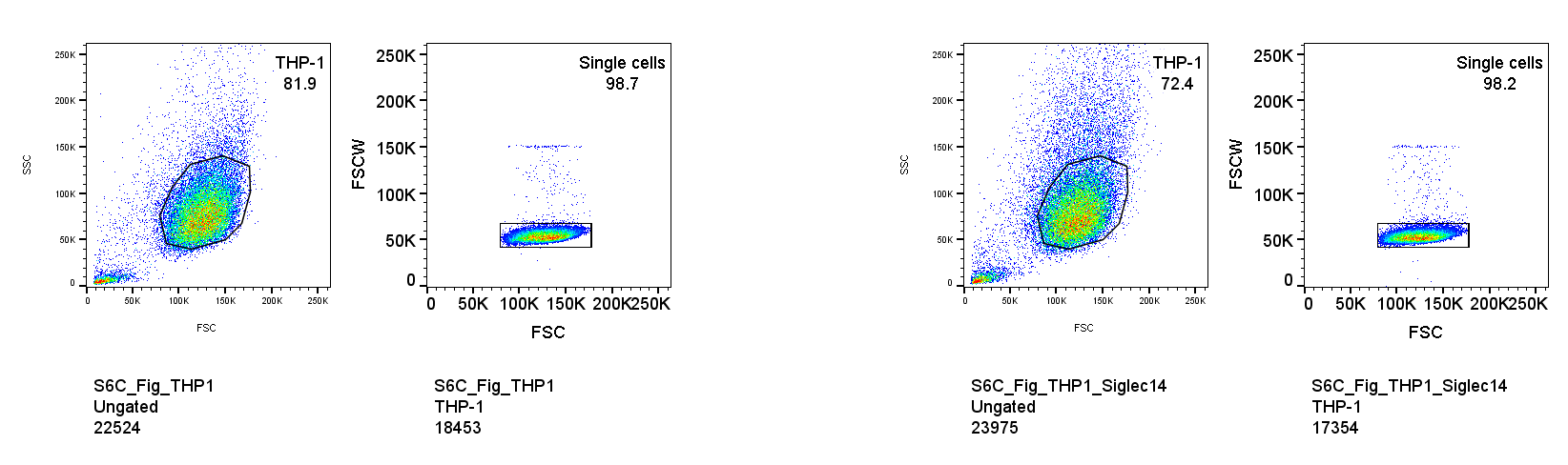

Supplement: S2 Data — (ZIP) [file pbio.3000549.s009.zip › S2_Data/S6C_Fig_Gating_strategy.tiff]

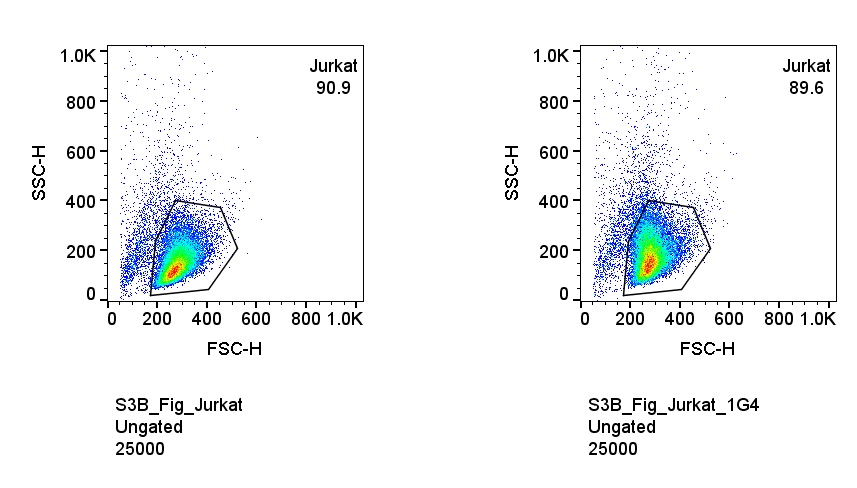

Supplement: S2 Data — (ZIP) [file pbio.3000549.s009.zip › S2_Data/S3B_Fig_Gating_strategy.tiff]

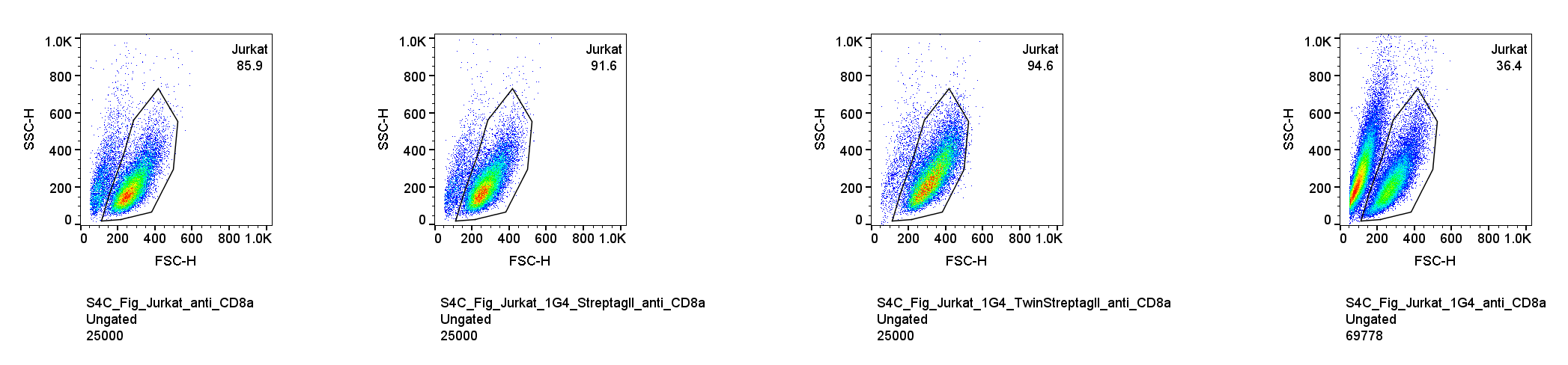

Supplement: S2 Data — (ZIP) [file pbio.3000549.s009.zip › S2_Data/S4C_Fig_anti_CD8a_Gating_strategy.tiff]

S1C Fig.

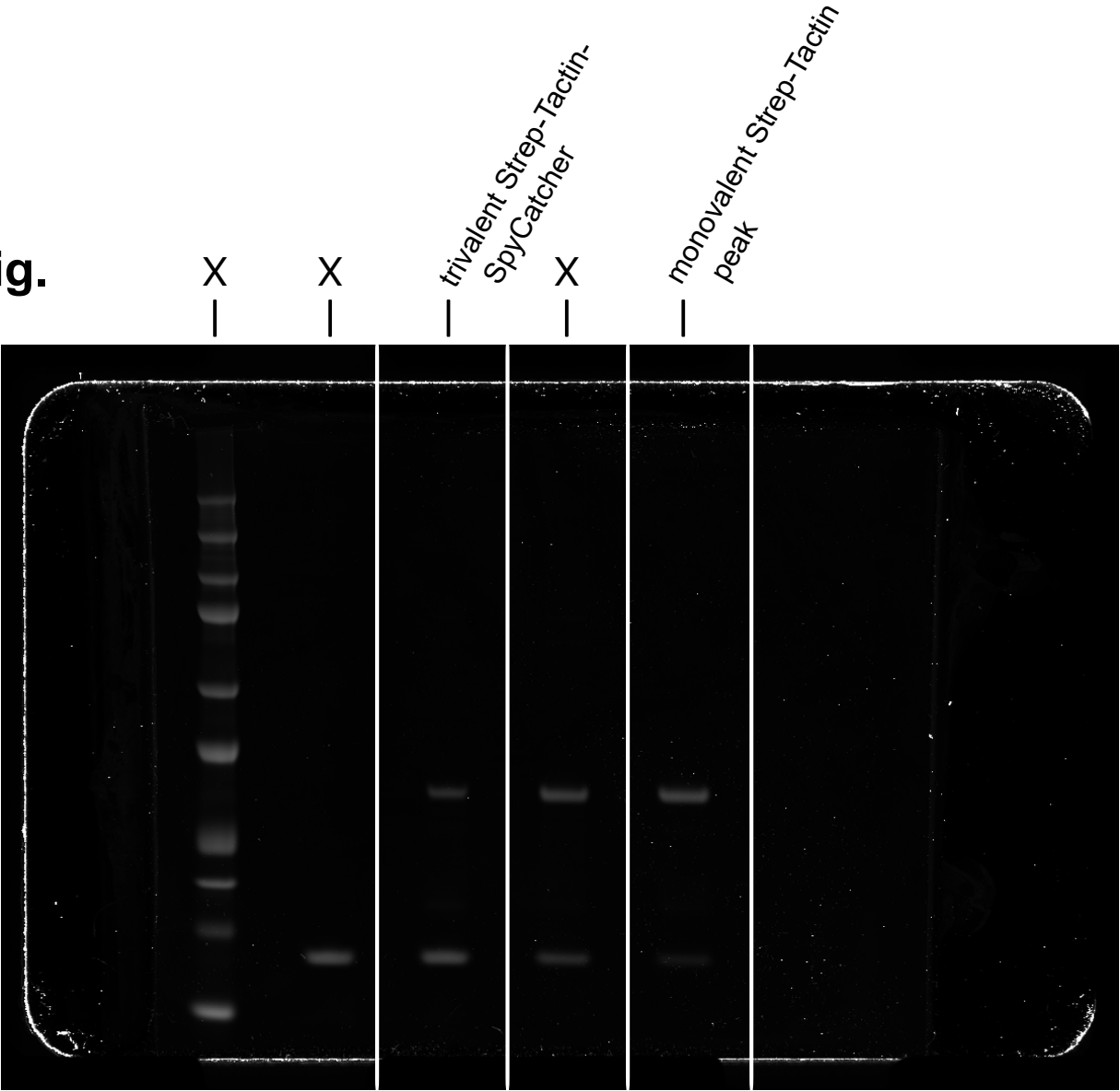

**S2D Fig.**

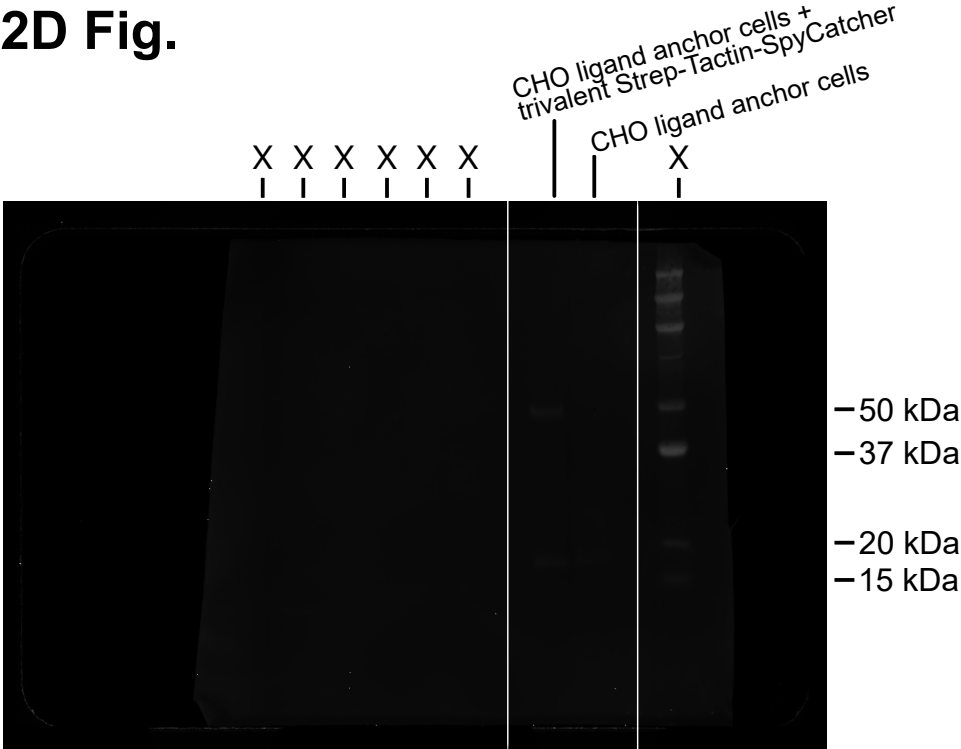

**IB: anti-HA tag**

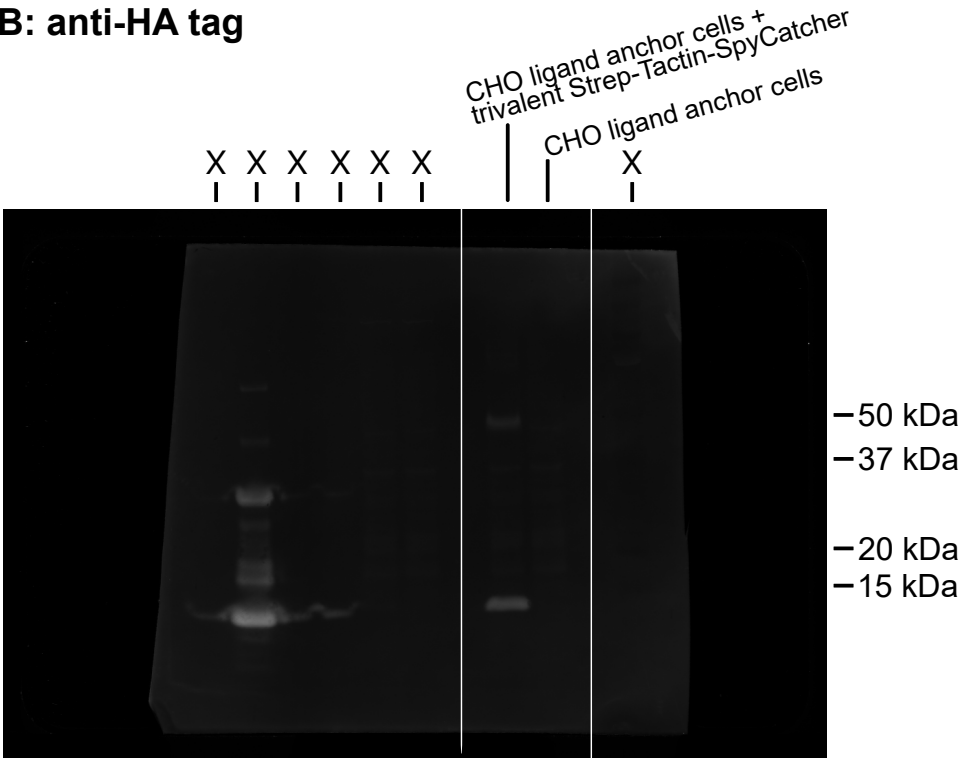

**IB: anti-streptavidin**

**S5D Fig.**

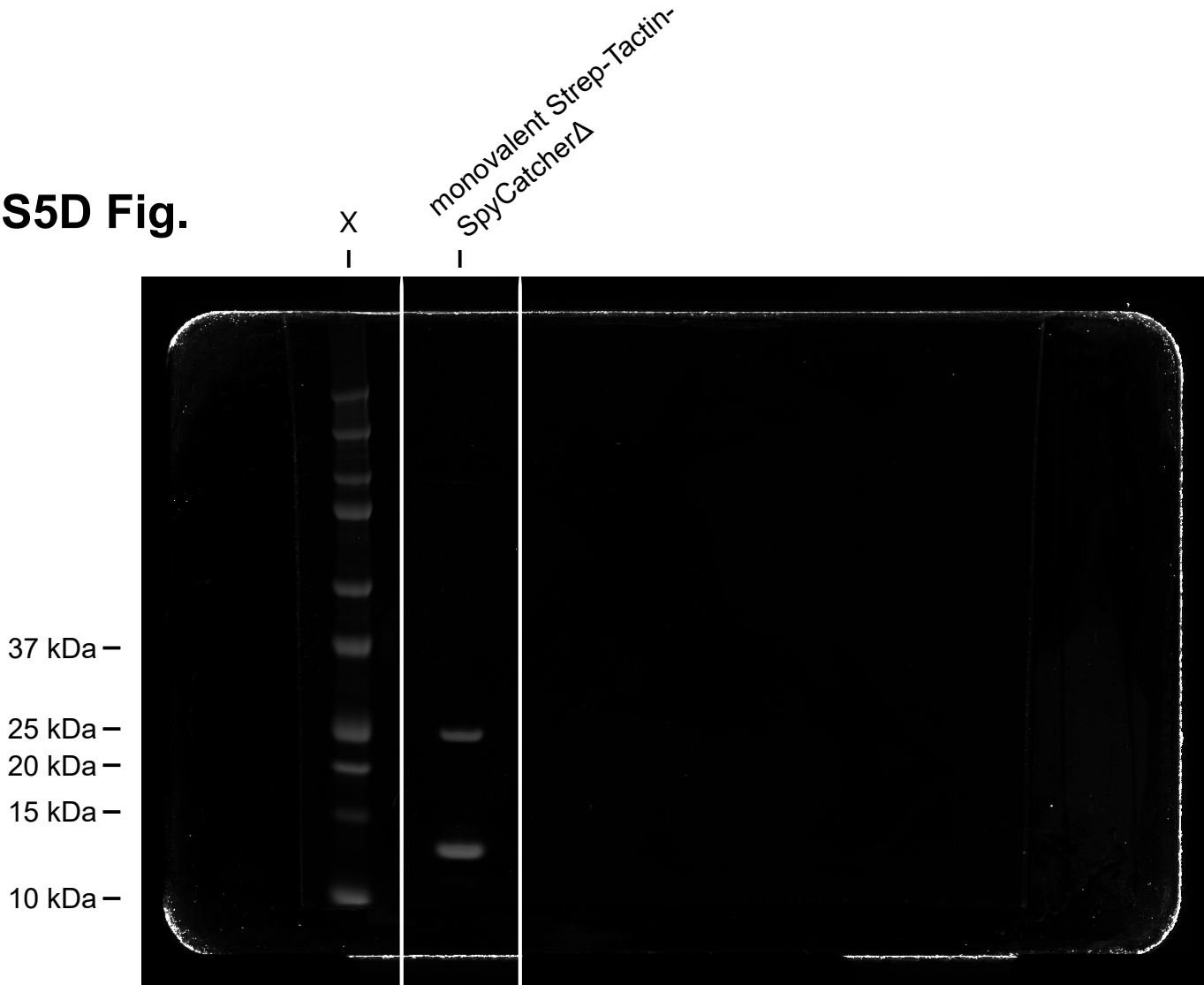

Supplement: S1 Raw images — Vertical white lines indicate where images were spliced to remove irrelevant lanes. Image colours were inverted and brightness of entire image as a whole was altered prior to creating figures. (PDF) [file pbio.3000549.s010.pdf]
