# Supplementary material for: A generic cell surface ligand system for studying cell–cell recognition
Source: PLoS Biol. 2019 Dec 9;17(12):e3000549. doi: 10.1371/journal.pbio.3000549 (PMC6922461; doi:10.1371/journal.pbio.3000549)
Supplement: Attachment — Submitted filename: Reviewer reply.docx [file pbio.3000549.s011.docx]

Reviewer #1: 

The authors describe a new way to generically engage cell surface receptors. The advantage of this approach is that the ligand can be incorporated into a target cell at a different density. The disadvantage is that the target receptor does need to be modified genetically and ectopically expressed. However, the authors demonstrate that this disadvantage is not a huge concern by showing that the modified protein recapitulating the natural response to a native ligand is similar to the artificial/universal ligand. 

While I think this is a very nice universal approach that could find usefulness, I do have one concern that relates to the last Figure of the paper. In this experiment, the authors express a receptor (Siglec-14) with only 1 StepTagII, or than 2 copies of the tag. They conclude that the cells were stimulated less with the version that contains 1 tag, which is a reflection of the 6-fold difference in dissociation constants between the monomeric vs dimeric interaction. However, this in the context of a synapse between two cells, where there are likely thousands of cell being engaged simultaneously. Therefore, what could equally be responsible for the differences are modest differences in expression levels between the two constructs. Did you authors check Siglec-14 expression levels (could be done with an anti-Sigelc-5 antibody)?

We thank the reviewer for their comments. As the reviewer rightly points out, differences in receptor expression levels could contribute to the differences the ligand density required for triggering. We have modified the text to acknowledge this important point. The main purpose of this experiment is to illustrate that we can manipulate the affinity and get stimulation with a substantially lower affinity interaction. Further studies are in progress to explore the relationship between affinity and the surface density required for triggering. In these experiments we will ensure that we precisely match receptor expression levels.

-------------


Reviewer #2: 

Denham et al. have developed a versatile approach to control receptor:ligand engagement making it theoretically easier to study the effects of various properties of receptor:ligand engagement (e.g. surface density, valency, dimensions, and affinity) on signaling output. This approach clearly has unique advantages compared to other systems people have used for control of receptor:ligand engagement. Others have used heterodimerization systems (e.g. FKBP:Frb + Rapalog) (James JR, Vale RD, Nature 2012, fig 5 and James JR. Science Signaling. 2018), but have not applied the approach to systematically study different receptor types with precision. Furthermore, such systems do not allow for easy manipulation of affinity or valency, and FKBP and Frb may be more problematic to fuse given their size (small tags are used here). Other approaches such as light controlled receptor ligand engagement (Baaske J. Comm. Biol. 2019 or [https://www.biorxiv.org/content/10.1101/432864v2.article-info](https://www.biorxiv.org/content/10.1101/432864v2.article-info)), allow for precise control of dwell time and receptor occupancy, but there a variety of complexities to the system that make it more difficult to take advantage of compared to the approach described here. The opto-proteins are often hard to work with, the approach requires special equipment, and fusion of the opto-proteins may not be straightforward. This approach also allows for control of parameters such as valency by simply changing the characteristics of the ‘bridging molecule’, Streptactin-Spycatcher, which is ideal.

While I agree that the approach described in the manuscript is versatile, I remain skeptical that it reliably mimics the natural receptor ligand interactions. The IG4 TCR data is somewhat convincing, but there is a significant difference in EC50. To me, 7,000 and 17,000 ligands/per cell is a big difference. Or at least it warrants providing data for at least one other receptor:ligand system (see below).

We are not claiming that the system mimics the natural ligand exactly. What we argue is that it is a reasonably proxy. While the reviewer is correct that there is a difference it is important to stress that these are mean values of data that are quite variable. We now provide raw data (Fig. 4d) to make clear the variation in the individual experiments.

Overall, I think the approach is useful and warrants publication if my major comments are addressed. The statistics, supplement, and description of methods are all sufficient.

MAJOR COMMENTS:

1)      Perform a similar set of experiments to what was done with the IG4 TCR with one of the other receptor systems (e.g. NKp30 and B7-H6) and see if the natural ligand data is in agreement with the generic ligand system.

This is an excellent suggestion but unfortunately runs up against a key problem that this generic ligand system was designed to tackle. Titrating the native ligand for NTR receptors is extremely challenging for the reasons we point out in introductory paragraph (lines 26-34). Indeed the only receptor with an easily titratable ligand is the TCR because of the ability to pulse peptides (although measuring pMHC surface density is difficult).

We have now included data using two chimeric antigen receptors (Fig. 6) to illustrate how our strategy can be used to titrate other ligands on cell surfaces. We are currently developing this approach so that we can titrate native ligands, but this is beyond the scope of the current paper.

2)      Provide data on changing valency for one of the receptor systems in figure 5. Since this is a methods and resources paper, I think it warrants showing data on versatility of the system. Changing valency is an important feature and would show one of the major benefits of this approach.

This is an excellent suggestion. We have now added data showing the effect of changing valency from monovalent to trivalent interactions (Figure 5d-f). Together with the new CAR data showing coupling of completely different ligands (Figure 6) we believe this amply demonstrates the versatility of our method.

3)      Can you shut off receptor:ligand engagement by titrating in a trivalent Strep-tactin without Spycatcher (especially when using a lower affinity Streptactin mutant)? It would be useful to show that one could do different durations of engagement at least on the minute to hour timescale. You could also use other approaches for cessation of receptor:ligand engagement like putting a protease site between Strep-tactin and Spycatcher (e.g. a TEV site).

This is another good suggestion, but we do not think it would be as interesting in long term assays as in short term assays such as those involving imaging (e.g. changes in motility, shape, and intracellular calcium). While the generic ligand system is well suited to such assays, we are not experts in these methods, and so this will require collaboration, and is beyond the scope of the current paper.

MINOR COMMENTS:

1)      Modify Figure 5 panels a, b, and c so receptor and ligand are visible

We have modified the figures so that the labels are more visible.

-------------


Reviewer #3: 

The authors presented a generic ligand and receptor system to study cell-cell interaction.  This system consists of a receptor that is engineered with a Strep-tag and a 2-component ligand system involving a spy-tag.  Functionally, this system is reminiscent of the FKBP/FRD system developed by John James to study CAR and TCR signaling (Sci Signaling, 2018), and split CAR designs developed by several groups (e.g., Rogers et al, PNAS, 2016 113 (4) E459-E468, Ma et al, PNAS, 2016 113 (4) E450-E458).  The authors failed to acknowledge these manuscripts.  The advantage of these systems is that, by varying the concentration of the soluble adaptor (either the Strep-Tactin-SpyCatcher from this manuscript or rapalog in John James’ system), one can regulate the activity of the receptor.  However, this feature has been demonstrated in the literature many times.  The only novel feature of the system presented by this manuscript is that the spy-tag system forms a covalent bond with the
ligand anchor.  This review cannot find any reason to believe that this feature is essential.
The downside of using an artificial ligand is that you lose the information on the real ligand-receptor interaction.  As noted by the authors, the geometry of the receptor interaction is important.  While this synthetic system will provide an orthogonal means of study such effect, ultimately, it is dangerous to draw too much conclusion from such a system to the real ligand-receptor interaction. 
While the system is well characterized, it represents yet another inducible receptor system that is titratable with a soluble ligand.  There is no new concept presented, just rephrased differently than other published work.  This work is publishable, but the novelty is unfortunately negligible.

We thank the review and agree that more care should have been taken in describing how our method compares with other titratable generic cell surface ligand systems already reported. We have now included discussion of these other methods, such as the FKBP/FRD and split CAR systems (see lines 293-304).

There are several things that that set our method apart from these:

(1) Firstly, several properties of our ligand other than surface density, including receptor-ligand affinity, valency, and size, can be relatively easily varied in our system. To more clearly illustrate these points we have added an example of varying the valency (Fig 5d-f)

(2) Secondly our method allows precise control and accurate quantification of the number of ligands. This is critical for studying the effects of varying ligand properties as the effects will be seen as changes in the density of ligand required for stimulation. An ability to precisely control and measure surface density also allows us to study what features of receptors and downstream signaling pathways affect the sensitivity and specificity of cell-cell-recognition. These are crucial features of cell-cell recognition that are poorly understood and are particularly important when engineering new synthetic receptors such as CARs.

(3) Finally, our system allows multiple ligands to be easily added to cells in controlled ways, greatly facilitating the analysis of signal integration.

We have now included more discussion of the advantages of our system in lines 305-312.

To help illustrate the points 2 and 3 we have added a final section, including a new Figure (Fig 6), describing our adaptation of the system to study two CARs that bind to two different ligands.

Although the reviewer downplays the importance of covalent attachment of ligands to the cell surface this is a significant advantage compared with soluble bifunctional bridging systems. In particular covalent attachment allows accurate quantitation of binding site number. For bispecific soluble adaptors the ligand density is a complicated relationship between the affinity of the soluble ligand for the ligand anchor, the number of ligand anchors and the concentration of soluble ligand added. With covalent attachment it is also clear how the surface density changes with time (see Supplementary Figure 2E)

We believe these characteristics allow this method to be of wider use and represent a significant improvement compared with previously published systems.

-------------

Reviewer #4: 

In this beautifully written paper, Denham and colleagues describe a method to quantitatively control ligand densities on cells in order to investigate cell signaling quantitatively in a more biological context than is typically performed. What is novel is the precision through which ligand density can be quantified and manipulated. Although some might argue for certain interactions this can be achieved via other means, these will be case dependent, and the method here is not. It is a clever and creative approach. A major caveat though is the receptor-ligand interaction is artificial - the receptor consists of a modified form, and the ligand will not be the real ligand. The authors pitch is that the goal is to study biological *signaling* processes, rather than receptor-ligand interactions, and that is fair. However, in many cases the two are intertwined. The T Cell Receptor interaction which they choose to use as a demonstration system is a good example. They propose the ability aspects such as clustering, etc., but there are ongoing questions as to whether features such as binding orientation and mechanical forces impact T Cell signaling mechanisms. Indeed these things could potentially underscore the differences in EC50 values they find for their constructed system with the TCR versus the actual TCR/pepMHC system they compare it to (lines 220-ish). Thus while the system is creative and clever and certainly more quantitative than some other approaches, there is a built-in limitation and could impact biological conclusions, at least for T Cells. Other cell signaling systems are likely to be less complex, but when it comes to things like clustering and valency, which the authors emphasize, the non-native ligand will be a point for argument. This does limit enthusiasm.

While the author is correct that there is, by definition, a difference between our generic ligand and the native ligand, we think that this should be considered an **advantage** of the system, for two reasons.

Firstly, it allows us to establish whether there are structural details of a particular interaction (such as conformation of the binding site or orientation) that are critical for recognition and signaling. Our finding that a generic ligand TCR ligand can trigger with similar surface density as a native ligand (FIG. 4c&d) argues against this, but clearly further detailed studies comparing downstream signaling and function will be needed in order to exclude subtle differences. Such an analysis REQUIRES a generic ligand system that can be manipulated as we can ours. We are currently adapting the system to enable us to also present native ligands in a controlled and titratable way. Comparing native and generic ligands will enable us to address the importance of conformation/orientation associated with native ligand binding for a wide range of receptors.

Secondly, our system, by utilizing exactly the same interaction to compare different receptors will allow us to compare different receptors without the confounding differences in ligands and receptor/ligand binding properties. Thus we can precisely compare the sensitivity of receptors as well as determine whether they differ in ability to discriminate between ligands of different affinity. The sensitivity and specify of cell surface receptors such as the TCR, and how these crucial properties vary between receptors, are important open questions. They have been refractory to investigation because of the absence of suitable tools, such as the system we describe here. Indeed, we have preliminary data in our system indicating that the TCR is not only more sensitive that other NTRs (Fig 3f) but also shows better discrimination between ligands of different affinities. Our generic ligand system will allow us to dissect the mechanisms underlying these interesting differences.

Some more specific comments:
1) Although the authors have all the sequences and constructs in the methods, the twin-step tag should be described in the results as the receptor will always be modified with this.

We have now added the sequence of Streptag II to the results section (line 71).

2) The authors need to perform binding experiments with purified protein to show that the twin-step tag does not interfere with binding to the pepMHC, or quantify the amount that it does (lines 225-ish).

We tested this by measuring by flow cytometry pMHC tetramer binding to T cells expressing TCRs with or without the twin Strep-tag II (Supplementary figure 4c). Binding was similar, indicating that the twin Strep-tag II did not interfere with pMHC binding.

3) Page numbers please.

Page numbers are provided.
