# Supplementary material for: A generic cell surface ligand system for studying cell–cell recognition
Source: PLoS Biol. 2019 Dec 9;17(12):e3000549. doi: 10.1371/journal.pbio.3000549 (PMC6922461; doi:10.1371/journal.pbio.3000549)
Supplement: Attachment — Submitted filename: Reviewer comments_responses.docx [file pbio.3000549.s013.docx]

Reviewer #2:

The authors were able to address my major concerns.

1) They have convinced me that a generic ligand can be informative even it it does not mimic aspects of the natural receptor:ligand geometry. This approach as with other systems like it can decouple the potential signaling capability of the receptor from the natural binding constraints. This is useful in synthetic biology where there is a desire to co-opt and engineer natural systems for therapeutic purposes.

They also point out that having a generic ligand system can provide a clue to whether the natural binding geometry and dynamics are a major contributor to the overall signaling and cellular outcome. For example, in the hypothetical case where their generic ligand system exhibits major differences from what has been observed for the natural system. This would suggest the natural binding features are critical to the biology. However, they haven't provided a case of this here.

2) They have showcased the versatility of their approach by providing data on the effects of ligand valency in figure 5.

3) I find their chimeric antigen receptor work interesting, but the data is minimal. This is a methods paper so I am okay with this. Overall, the approach could be interesting to use to study different types of signaling domains individually or in concert.

In the end, this approach may be most useful for synthetic biology purposes where we seek to simultaneously understand how we can gain control over activation properties of a receptor system and at the same time be able to retarget the receptor to any ligand of interest. I would suggest adding a passage on this to manuscript.

We agree that the system we present is useful for synthetic biology approaches. We have modified the text to more explicitly describe such applications. See lines 790-793.

--------------


Reviewer #3:

My biggest concern with this work is its differentiation over existing work, especially with regard to the split CAR systems that have been developed recently.  The author listed 3 things that set themselves apart from existing work.  However, most of the features that the authors highlighted as unique have been achieved elsewhere.

1.  The authors suggest that their system allows variation in receptor ligand affinity, valancey and size.  However, this can be all be achieved by split CAR design by changing the antibody affinity, number of antibody domain, or changing the size of the binding domain.  Please see Cho et al, Cell 2018 (ref 7 in this manuscript) for an example of ligand affinity modulation.  While these published articles didn't compare the size of the adapter molecules, I cannot see why this is not achievable with other systems.

We appreciate that the split CAR system described in Cho et al 2018 (ref 7 in our manuscript) could in principle be adapted to alter ligand densities and have added text to acknowledge as such (lines 783-787). We do however believe there are two disadvantages to using leucine zipper pairs in this approach. Firstly, the non-covalent interaction could complicate the ability to precisely quantitate and finely control receptor/ligand density through time. Secondly, the ability to alter valency by creating fusions of multiple antibody domains would require the ligand to be split in two by means of a leucine zipper pair as opposed to the receptor as done by Cho et al for the split CAR system. Creating such a “split ligand” via this method is possible but, we feel, distinct from the split CAR system already described.

2.  The authors suggest that their system affords accurate quantification of the number of ligands, implying that there are not other methods.  NIST has been developing standards using antibody and flow cytometry to quantify surface molecules. This is now the standard approach to obtain absolute measurement of surface molecules.  It is hard for me to agree that the author's approach is better than what NIST has been developing, especially considering the serious restriction that this system impose over the use of fluorescent-labeled antibody.

Cytometry A. 2012 Jul;81(7):567-75. doi: 10.1002/cyto.a.22060. Epub 2012 Apr 26.
Human CD4+ lymphocytes for antigen quantification: characterization using conventional flow cytometry and mass cytometry.
Wang L1, Abbasi F, Ornatsky O, Cole KD, Misakian M, Gaigalas AK, He HJ, Marti GE, Tanner S, Stebbings R.

It was not our intention to imply the method we present is the only method available for cell surface protein quantitation. We argue that the surface density of ligands has not been both varied and quantified in any of the previous studies we cite that permit flexible control over the abundance of receptor-ligand interactions. And thus our system offers an advantage over those studies. We have modified the text (lines 733-736) to be more explicit and clarify our meaning.

3.  The authors suggests that their system affords multiple ligands to be easily added.  Again, please see Cho et al, Cell, 2018 for a published example of combinatorial split CAR system that targets 2 ligands in human primary T cells.

We have added text (lines 799-802) to acknowledge there are other approaches that could be used to present multiple surface ligands including the specific reference provided by the reviewer.

Based on these facts, I am still not convinced that this work is sufficiently novel over existing work.  The major difference between this work and existing split CAR design is the covalent linkages vs. antibody binding (which can have a very high affinity that approaches covalent linkages).  The authors imply that covalent linkages afford unique properties that other systems cannot achieve, such as modulation in valency, ligand affinity, and quantification of ligand density, which is patently false.

We believe the strength of using a covalent interaction to join two components of the ligand is that it simplifies the variation, and quantitation, of ligand density. While we acknowledge that leucine zipper pairs can have high affinities, using reversible interactions could arguably complicate the precise quantitation of ligand density and maintenance of that density throughout an experiment.
